# Protocol for a randomized clinical trial investigating early sacral nerve stimulation as an adjunct to standard neurogenic bladder management following acute spinal cord injury

**DOI:** 10.1186/s12894-018-0383-y

**Published:** 2018-08-29

**Authors:** Jeffrey D. Redshaw, Sara M. Lenherr, Sean P. Elliott, John T. Stoffel, Jeffrey P. Rosenbluth, Angela P. Presson, Jeremy B. Myers, for the Neurogenic Bladder Research Group (NBRG.org)

**Affiliations:** 1 Department of Surgery, Division of Urology, University of Utah School of Medicine, 30 N. 1900 E. 3B110, Salt Lake City, UT 84132 USA; 2 Department of Urology, University of Minnesota, Minneapolis, MN USA; 3 Department of Urology, University of Michigan, Ann Arbor, MI USA; 4 University of Utah School of Medicine, Physical Medicine and Rehabilitation, Salt Lake City, UT USA; 5 Division of Epidemiology, University of Utah, Salt Lake City, UT USA

**Keywords:** Sacral neuromodulation, InterStim, Neurogenic bladder, Spinal cord injury, Bladder

## Abstract

**Background:**

Neurogenic bladder (NGB) dysfunction after spinal cord injury (SCI) is generally irreversible. Preliminary animal and human studies have suggested that initiation of sacral neuromodulation (SNM) immediately following SCI can prevent neurogenic detrusor overactivity and preserve bladder capacity and compliance. We designed a multicenter randomized clinical trial to evaluate the effectiveness of early SNM after acute SCI.

**Methods/Design:**

The scientific protocol comprises a multi-site, randomized, non-blinded clinical trial. Sixty acute, acquired SCI patients (30 per arm) will be randomized within 12 weeks of injury. All participants will receive standard care for NGB including anticholinergic medications and usual bladder management strategies. Those randomized to intervention will undergo surgical implantation of the Medtronic PrimeAdvanced Surescan 97,702 Neurostimulator with bilateral tined leads along the S3 nerve root in a single-stage procedure. All patients will undergo fluoroscopic urodynamic testing at study enrollment, 3 months, and 1-year post randomization. The primary outcome will be changes in urodynamic maximum cystometric capacity at 1-year. After accounting for a 15% loss to follow-up, we expect 25 evaluable patients per arm (50 total), which will allow detection of a 38% treatment effect. This corresponds to an 84 mL difference in bladder capacity (80% power at a 5% significance level). Additional parameters will be assessed every 3 months with validated SCI-Quality of Life questionnaires and 3-day voiding diaries with pad-weight testing. Quantified secondary outcomes include: patient reported QoL, number of daily catheterizations, incontinence episodes, average catheterization volume, detrusor compliance, presence of urodynamic detrusor overactivity and important clinical outcomes including: hospitalizations, number of symptomatic urinary tract infections, need for further interventions, and bowel and erectile function.

**Discussion:**

This research protocol is multi-centered, drawing participants from large referral centers for SCI and has the potential to increase options for bladder management after SCI and add to our knowledge about neuroplasticity in the acute SCI patient.

**Trial registration:**

ClinicalTrials.gov #NCT03083366 1/27/2017.

## Background

Urinary bladder dysfunction and incontinence have a significant clinical, physical, and quality of life (QoL) burden in patients with spinal cord injury (SCI). Contemporary studies report bladder problems are the second leading reason SCI patients seek medical care. Almost 80% of SCI patients report some degree of bladder dysfunction within 1 year of injury and 42% are hospitalized for urinary problems every year [1–4]. Renal failure and urinary sepsis historically were the major causes of death in SCI patients after recovery from the initial injury [5]. Advances in urologic care, specifically the introduction of clean intermittent catheterization (CIC) in the 1970’s revolutionized the care of SCI patients [6, 7]. However, there is a significant inconvenience, potential dependence on others, and often continued leakage that leads to patient non-compliance and discontinuation of CIC [4, 8, 9]. The currently established goals of neurogenic bladder (NGB) management include; prevention of renal insufficiency or failure by keeping bladder pressures low, preservation of urinary continence, and optimization of QoL. Current available treatments including pharmacologic therapy, injection of botulinum toxin, and surgical bladder augmentation or urinary diversion all address NGB physiology at the bladder level rather than the neurologic injury leading to NGB.

Largely, NGB cannot be reversed and prevention of the development of some of the worst aspects of NGB such as poor compliance, high intravesical filling pressures, and spasticity by trying to address the neurologic cause of these sequelae is a unique research approach. Over the past 20 years sacral neuromodulation (SNM) has become an established treatment for refractory urinary urge incontinence, urinary frequency/urgency syndrome, non-obstructive idiopathic urinary retention and chronic fecal incontinence [10–13]. The surgical procedure is minimally invasive and has few risks. Recent animal and human pilot data suggest that SNM implemented in the acute setting after SCI may preserve bladder compliance, bladder volume, and reduce urinary tract infections [14–16].

Based on this encouraging preliminary data, we designed a prospective, randomized, multicenter clinical trial to assess the efficacy and safety of SNM for treating neurogenic bladder dysfunction in patients with spinal cord injuries. The study hypothesizes that sacral neuromodulation, initiated during the acute phase following spinal cord injury, can decrease bladder spasticity preserving bladder compliance, bladder volume, and low bladder filling pressures. This will result in improvements in both objective quantifiable clinical outcomes, as well as subjective patient reported quality of life compared with standard neurogenic bladder management.

## Methods and design

### Study design

Patients will be randomized to standard neurogenic bladder management or standard management plus sacral neuromodulation (via S3 nerve root stimulation) in a parallel non-blinded fashion with the intent to demonstrate the superiority of sacral neuromodulation plus standard management over standard management alone. Patients randomized to the intervention arm, will undergo implantation of the PrimeAdvanced Surescan 97702 Neurostimulator (Medtronic, Minneapolis, MN) with bilateral tined leads within 12 weeks of spinal cord injury.

### Study locations

The study will take place at the Universities of Michigan, Minnesota, and Utah. All three of these sites are large volume academic hospitals that see a high volume of patients with SCI and are associated with acute rehabilitation centers that treat SCI immediately after hospitalization from injury.

### Study population and recruitment

Patients will initially be identified with the assistance of our colleagues in either Physical Medicine & Rehabilitation or Neurosurgery according to the inclusion and exclusion criteria in Table 1. All consecutive patients will be eligible for screening and approached for enrollment.

**Table 1:** Study inclusion and exclusion criteria

Inclusion criteria	
Age > 18 years	
Ability to implant device less than 12 weeks post-SCI	
Presence of acute SCI at or above T12	
ASIA scale A or B	
Expectation to perform CIC personally or have caretaker perform CIC	
Medically stable to discharge to a rehab setting	
Exclusion criteria	
Inability to perform CIC or have caregiver perform it	
Pre-existing SCI	
Pre-existing progressive neurological disorder	
Autonomic dysreflexia	
Prior sacral back surgery	
Posterior pelvic fracture with distortion of the sacroiliac joint	
Prior urethral sphincter or bladder dysfunction	
Chronic urinary tract infections prior to SCI	
Pregnancy at the time of enrollment	
Presence of coagulation disorder or need for anticoagulation that they connot be stopped temporarily for procedure	
Any significant co-morbidity or illness that would preclude their participation or increase the risk to them having a surgical procedure	
Active untreated infection	
Traumatic injury to the genitourinary system	
Prior pelvic radiation, bladder cancer or other surgical procedure to the bladder that would effect baseline bladder physiology	

### Investigations

**Fig. 1:**
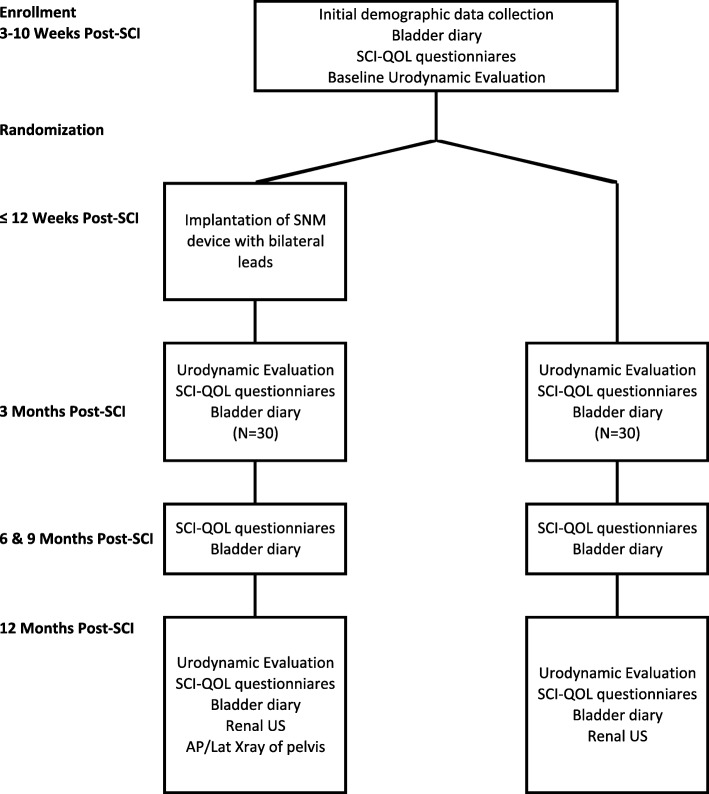
Summary of study protocol. Spinal cord injury (SCI), Sacral neuromodulation (SNM), Neurogenic bladder (NGB), S3 Sacral nerve root (S3), Quality of life (QoL)

Sixty patients will be enrolled and randomized (30 per arm). A timeline and an overview of the procedures and diagnostic studies that will occur during the course of the study are shown in Fig. 1. The PrimeAdvanced Surescan 97702 Neurostimulator (Medtronic, Minneapolis, MN, USA) with bilateral tined leads will be implanted in patients randomized to the intervention arm using a standard surgical technique. Because the study hypothesis dictates early stimulation of the sacral nerve roots with a primary outcome evaluated 1 year post implant, the quadripolar tined leads and pulse generator will be placed in the same procedure. Food and drug administration (FDA) approved programming parameters for the InterStim II system will be utilized in this study.

All patients will receive usual standard of care bladder management for neurogenic bladder. Specifically, standard treatment entails the following: [1] clean intermittent catheterization (CIC) at regular timed intervals, [2] treatment with anticholinergic medicine or botulinum toxin as indicated to increase bladder compliance, decrease urinary leakage, and lower bladder pressures to prevent renal damage, [3] routine follow up in the urology and physical medicine and rehabilitation clinic [17].

At enrollment, all participants will complete the following: [1] standardized 3-day voiding diary to annotate the catheterization time, amount and time of fluid intake, and incontinence events, [2] 24 h pad weight test, [3] 3-day bowel diary to quantify baseline bowel habits, [4] inventory of current medications, [5] the Neurogenic Bladder Symptom Score (NBSS), [6] Spinal Cord Injury Quality of Life questionnaire (SCI-QoL) Bladder Management Difficulties, [7] SCI-QoL Bladder Complications, [7] the Neurogenic Bowel Dysfunction Score, [8] a non-validated questionnaire about autonomic dysreflexia impact, [9] Sexual Health Inventory for Men (SHIM), and [18] baseline urodynamic testing. Repeat measurements, other than urodynamic testing, will be obtained during clinic visits at 3, 6, 9, and 12 months after enrollment. For patients randomized to the intervention arm, urodynamic testing will be completed less than 2 weeks before implantation of the device. Urodynamic testing will be repeated at 3 months and then again at 12 months.

At each time point, a retrospective review since the prior time point will be performed in order to capture major events such as urinary tract infection (UTI), hospitalizations, additional surgery, as well as additional subjective data. Those additional subjective data will include: (1) current usage of anti-cholinergic medications and/or botulinum toxin treatment (2) bowel program including use of medication and mechanical aids (i.e. digital stimulation, enemas, etc.), (3) use of medications for sexual function, (4) symptomatic UTIs requiring antibiotic treatment, (4) complications attributable to the device, (5) need for revision of device or leads. A renal ultrasound will be obtained at the end of 12 months to evaluate for the development of hydronephrosis. This will be compared with baseline CT or US imaging obtained during their initial trauma evaluation. Serum creatinine as an estimate of renal function will be collected annually. Variables to be collected during the course of the study and timeline for collecting them is shown in Table 2.

**Table 2:** Primary and secondary outcome variables. UDS (urodynamic study), NBSS (Neurogenic Bladder Symptom Score), SCI-QoL (spinal cord injury quality of life measurement system bladder management difficulties and bladder complications), SHIM (sexual health inventory for men)

	Collection method	Time points (month)	Variable type	Analyzed At
Urodynamic parameters – aim 1				
Primary				
Maximum cystometric capacity	UDS	0,12 m	Continuous	12 m
Secondary				
Maximum cystometric at 3 months	UDS	0,3 m	Continuous	3 m
Bladder compliance	UDS	0,3,12 m	Continuous	3 m, 12 m
Presence of detrusor overactivity	UDS	0,3,12 m	Binary	3 m, 12 m
Volume & pressure at first detrusor contraction	UDS	0,3,12 m	Continuous	3 m, 12 m
Quality of Life – Aim 2				
Primary				
Difference in mean NBSS, SCI-QoL questionnaires	Questionnaire	3,6,9,12 m	Continuous	3,6,9,12 m
Secondary				
Daily number of catheterizations,	Bladder diary	3,6,9,12 m	Continuous	3,6,9,12 m
Average catheterization volume,	Bladder diary	3,6,9,12 m	Continuous	3,6,9,12 m
Urinary incontinence episodes per day	Bladder diary	3,6,9,12 m	Continuous	3,6,9,12 m
24 h pad weight test	24 h pad	3,6,9,12 m	Continuous	3,6,9,12 m
Clinical – Aim 3				
Primary				
# of UTIs requiring antibiotics	Chart review	12 m	Continuous	12 m
Secondary				
Development of hydronephrosis	Renal ultrasound	12 m	Categorical	12 m
Need for anticholinergic medication	Chart review	12 m	Binary	12 m
Botulinum toxin injection	Chart review	event	Binary	12 m
Need for device revision	Chart review	event	Binary	12 m
Device explanation	Chart review	event	Binary	12 m
Use of medications / mechanical bowel stimulation	Chart review	event	Continuous	12 m
SHIM	Chart review	event	Continuous	12 m
Erectile dysfunction medications	Chart review	event	Continuous	12 m

### Safety

The study safety monitoring committee will consist of a study investigator, a non-investigator urologist familiar with SNM, and an independent safety monitor (ISM) (a non-investigator familiar with randomized trial design). The safety committee will meet semi-annually and the ISM will have the authority to halt the study. Videos of the 1st procedures and two other randomly assigned procedures of the 10 expected from each center will be produced and viewed by the safety monitoring committee to assure consistency in placement of the SNM device. The following criteria will be used to evaluate the surgical technique used for the procedures.

*Video evaluation criteria for device implantation:*

- Maintenance of sterile procedure and use of a double prep with an iodine skin protective cover.
- Placement of electrodes appropriately using fluoroscopy in the S3 foramen.
- Adequate testing for motor response indicating close proximity of the nerve and appropriate placement in the S3 foramen.
- Correctly attaching electrodes to the IPG device and tunneling of electrodes.
- Appropriate closure of all incisions in a manner consistent with preventing erosions of the electrodes or generator.

Reportable safety concerns will be any of the following: (1) device infection requiring antibiotics or removal (significant rates triggering a full review and report will be greater than 5% infection rate), (2) revision of device and reason for revision accounting for surgical, device, or patient factors (significant rates triggering a full review of the device use will be greater than a 30% revision rate over the course of study), (3) autonomic dysreflexia rates in patients within both arms of the study as assessed by the autonomic dysreflexia specific questionnaires and any autonomic dysreflexia, which causes patients to turn off the device for more than a few minutes, not use the device as intended, or are the reason for explantation, (4) the need for removal of the device for erosion or pressure ulcers, (5) the need to remove the device due to the need for MRI imaging, (6) surgical complications within 6 weeks as classified by the Clavien-Dindo classification. Any events in category 1–6 will be reviewed by the study investigators as a whole during a semi-annual report from the safety committee. Event rates 1–6 will be disclosed to participants in the study during the consent process after they have been reviewed by the safety committee and investigators.

Safety committee will include reports to the FDA, institutional IRB’s, and to the funding agency (the Department of Defense) at 6 month intervals or as specified by the rules of each organization. A written report of all adverse events will be created every 3 months and a log maintained with study documents.

An interim analysis will be conducted at 50% enrollment (*N* = 26 after loss to follow-up, 13 patients per arm) accrual, and the decision to stop early will be governed by a significance level of 0.003. The ISM decision to stop early will be guided both by interim results and clinical judgment, especially in the context of emerging, relevant literature.

### Study device

The PrimeAdvanced Surescan 97702 Neurostimulator is an implantable device marketed by Medtronic (Minneapolis, MN, USA) for use in the United States. It is FDA approved for spinal cord stimulation rather than sacral nerve root stimulation, which is the intention of this study, however, it is conceptually and functionally, similar to the InterStim II Model 3058 neurostimulator, which is FDA approved for sacral neurostimulation in the United States. Importantly, unlike the InterStim II, the PrimeAdvanced has the ability to accommodate and simultaneously stimulate bilateral leads. All the preliminary studies in acute spinal cord injury have utilized bilateral sacral stimulation, which we believe offers a certain amount of redundancy defending against failure due to lead migration or malfunction and possibly increased efficacy.

Implantation is usually accomplished via a two-step process involving a placement of the permanent quadripolar electrode alongside the S3 nerve root under local or general anesthesia. This permanent lead is controlled by a temporary external programming device for 7–14 days. If greater than 50% improvement in clinical symptoms over that test period, the internal pulse generator (IPG) is implanted subcutaneously under during a second surgical procedure. Because the study hypothesis dictates early stimulation of the sacral nerve roots with a primary outcome evaluated 1 year post implant, the quadripolar electrical lead and IPG will be placed in the same procedure. This will avoid delay in stimulation of the sacral nerves during the critical window for preventing adverse neuroplastic changes. Patients will be mostly insensate due to their SCI or being under general anesthesia for the procedure. Therefore, to confirm intraoperative placement we will rely upon expected motor responses associated with S3 stimulation (anal bellows and toe flexion). If correct motor responses cannot be elicited intraoperatively than the patients will not undergo implantation and will be included in the randomized arm but in an ‘intention-to-treat’ manner.

FDA approved programming parameters for the InterStim II system will be utilized in this study as follows. The device will provide continuous stimulation (i.e. always on) without discrete treatment periods. The PrimeAdvanced Surescan IPG is capable of generating a maximum voltage of 10.5 V, 2 V higher than the InterStim II system, however, it will be programed to function within the normal InterStim II system parameters (maximum 8.5 V). Additionally, it will be programed to use the same stimulation pulsewidth (210 msec) and frequency (14 Hz) as the InterStim II system. The area under the stimulation curve will be the same or less than intended by the InterStim II parameters.

Each patient will be evaluated intraoperatively as is standard clinical practice to select stimulation parameters that result in typical, consistent physiological responses (e.g. anal bellows and first toe flexion). The default 14 Hz frequency will be maintained. The electrode with the lowest amplitude stimulation as a proxy for the most closely placed electrode to the S3 nerve. This electrode will be trialed first. The stimulation amplitude will be set at 0.7 V per protocol as was utilized in the one prior bilateral SNM study in acute SCI. Note: If the subject has S3 sensation (incomplete SCI), the amplitude will be set 0.1 V lower than the level of sensation. In such sensate patients, each lead will be programmed individually and then both leads will be activated simultaneously to determine whether further amplitude reduction is needed. These parameters will be set during the initial programming session and will remain constant for the duration of the study. All study centers will utilize the same protocol.

### Statistical analyses and outcomes

Sample size calculations for this study were based on the preliminary research by Sievert et al., and a feasibility of enrolling 30 patients per arm within the study period [16]. After accounting for a 15% loss to follow-up we expect 25 evaluable patients per arm. Using thresholds for study power of 80% (5% significance level) we would detect a 38% treatment effect (84 mL difference in bladder capacity).

#### Aim 1: To determine the effect of sacral neuromodulation on urodynamic parameters in the setting of acute spinal cord injury

The efficacy of SNM on the following urodynamic parameters will be evaluated at 3 months and 1 year post-injury: (1) maximum cystometric capacity, (2) bladder compliance, (3) presence of detrusor overactivity, and (4) volume and pressure for first detrusor contraction. Maximum cystometric capacity at 1 year is the *primary outcome*, and it will be compared between SNM and control arms using restricted maximum likelihood estimation under a linear mixed model controlling for institution [19]. Both an intention to treat and per-protocol analysis will be performed to account for any patients who were randomized to the intervention arm, but were unable to undergo placement of the device due to poor motor response intraoperatively.

#### Aim 2: To assess the impact of sacral neuromodulation on patient-reported quality of life after acute spinal cord injury

Patient-reported QoL will be assessed using the Neurogenic Bladder Symptom Score (NBSS) and the two bladder specific item banks from the spinal cord injury quality of life measurement system (SCI-QoL) Bladder Management Difficulties and Bladder Complications. We will use a similar linear mixed model framework as described in Aim 1 to compare the mean questionnaire scores at each follow-up assessment between the intervention (SNM) and control arms. *The primary outcome* will be the difference in mean questionnaire scores. *Secondary outcomes* will include daily number of catheterizations, average catheterization volume, and episodes of incontinence per day will be compared between groups. The primary efficacy will be evaluated at 1 year between the randomized groups. Secondary contrasts will evaluate intermediate treatment effects at earlier time points, and on average across all time points.

#### Aim 3: To examine the impact of sacral neuromodulation on quantifiable clinical outcomes

Patients will be followed longitudinally during the study period and assessed for the following: (1) number of symptomatic UTIs per year, (2) need for anti-cholinergic medications and/or botulinum toxin treatment, (3) complications attributable to the device, (4) need for revision of device or leads due to lead migration or failure, (5) development of hydronephrosis, (6) the need for medications and or mechanical aids (i.e. digital stimulation, enemas, etc.) for bowel program (7) SHIM scores, and (8) use of medications for erectile function. Our primary outcome will be the rate of symptomatic UTIs requiring antibiotic treatment over the 12-month study period. UTIs and many of our secondary complications can be experienced multiple times throughout the year. As a result, we will use a frailty model to handle complications that are potentially recurring events [20]. This method allows for heterogeneity among the evaluable subjects in terms of their differences in complication risk, as it is likely that some subjects will be more prone to UTIs (and other complications) than others. Events such as the use of anticholinergic medicines for control of detrusor overactivity are simply yes/no indicators during the annual period, and are thus more meaningfully modeled in the typical Cox regression framework for analyzing single rather than recurrent events. Again, all analyses will control for institution.

### Ethics and dissemination

An investigational device exemption (G160136/A002) was obtained from the FDA. A centralized IRB (based at the University of Utah) will approve a data and safety monitoring plan to the risks and complexity of this trial. Approval from the USAMRC ORP Human Research Protection Office has also been granted. This trial will be performed in accordance with the guidelines for Good Clinical Practice in Clinical Trials. Handling of all personal data will strictly comply with the Health Insurance Portability and Accountability Act. The trial has been registered at clinicaltrials.gov (https://clinicaltrials.gov/ct2/show/NCT03083366).

## Discussion

Sacral neuromodulation has a very well established track record in the treatment of patients with non-neurogenic urinary and fecal dysfunction. In the US, the InterStim Therapy System (Medtronic, Minneapolis, MN, USA) has been FDA approved for use in idiopathic overactive bladder since 1997, urinary frequency/urgency syndrome and non-obstructive idiopathic urinary retention since 1999, and for chronic fecal incontinence since 2011 [21–24]. Efficacy is well established for all three uses. In idiopathic overactive bladder, SNM achieves sustained therapeutic success in 85% of patients with a greater than 60% reduction in leaks per day. From a quality of life standpoint, 80% of subjects report significant improvement in their urinary symptoms [23]. Similar improvements with SNM are noted when treating chronic fecal incontinence, with 86% of patients achieving therapeutic success [25].

Once changes in the neurological control of the bladder have occurred following SCI they are in most cases irreversible. As a result, treatments must be directed at the local muscle level in order to control the high bladder pressures, incontinence and other symptoms. Consequently, the majority of research into the effects of spinal cord injury on the urinary bladder has focused on patients with well-established chronic neurogenic bladder physiology. Interventions during the acute phase of SCI aimed at preventing the development of, or reducing the symptoms of NGB, has not been extensively studied.

SNM implemented during the acute phase of SCI has good theoretical and experimental support in both animal and human clinical studies. In an animal model of complete spinal cord injury, Shi et al. demonstrated that SNM, could reduce peak bladder pressures and uninhibited detrusor contractions during bladder filling [14]. In vitro on a tissue level, in isometric relaxation experiments complete spinal cord transection caused a decrease in β-adrenergic relaxation responses which was shown to be muted by SNM in an animal model [15].

In humans, a pilot feasibility study has also demonstrated efficacy of SNM in the acute phase of SCI. Sixteen patients with traumatic complete SCI were enrolled during the acute bladder-areflexia phase [16]. Ten of these patients were implanted with bilateral sacral neuromodulators in a non-randomized fashion less than 3 months after their initial spinal cord injury. Six patients who met inclusion criteria, but did not wish to undergo treatment were used as controls. At 1 year follow-up, urodynamic studies showed an increased capacity (582 mL vs 294 mL), improved compliance and end filling pressures. The patients who underwent SNM had fewer UTIs (0.5/yr. vs. 3.8/yr) and hospital admissions. Other benefits of SNM included elimination of incontinence, in fact, none of the patients in the SNM group experienced incontinence, compared to 100% of the control group. Additional evidence for improvement in bladder pressures was the decreased need for anti-cholinergic medications (20% in the SNM group vs. 100% in controls).

This research protocol is multi-centered, drawing participants from large academic referral centers for SCI and has the potential to increase options for bladder management after SCI and add to our knowledge about neuroplasticity in the acute SCI patient.

### Trial status

The trial is in the recruiting phase at the time of manuscript submission.

## Abbreviations

CIC: Clean intermittent catheterization
FDA: Food and drug administration
IPG: Internal pulse generator
ISM: Independent safety monitor
NBSS: Neurogenic bladder symptom score
NGB: Neurogenic bladder
QoL: Quality of life
SCI: Spinal cord injury
SHIM: Sexual health inventory for men
SNM: Sacral neuromodulation
UTI: Urinary tract infection
